# Dr. Cooper’s Death

**Published:** 1906-09

**Authors:** 


### EDITORIAL NOTES AND COMMENTS.

The Business office of THE JOURNAL-RECORD is No. 1007-1008 Century Building.

The Editorial office is 318-319-320 The Prudential.

Address all Business communications to Dr. M. B. Hutchins, Mgr.

Make remittances payable to THE ATLANTA JOURNAL-RECORD OF MEDICINE.

On matters pertaining to the Editorial and Original Communications address Dr. Bernard Wolff, Atlanta.

Reprints of original articles will be furnished at cost price. Requests for the same should always be made *on the manuscript*.

We will present, post-paid, on request, to each contributor of an original article, twenty (20) marked copies of THE JOURNAL-RECORD containing such article.

### DR. COOPER'S DEATH.

AFTER an illness of three weeks, Dr. Hunter P. Cooper, one of the leading physicians and surgeons of the State, and a prominent citizen of Atlanta, died August 24th at his residence, 598 Peachtree street, aged forty-six. Dr. Cooper succumbed to an attack of meningitis complicated with nephritis. There were few more popular physicians in Atlanta than Dr. Cooper, who was closely allied with many of the leading institutions in the city. He was a graduate of the Medical Department of the University of Virginia, and afterwards studied in New York at the College of Physicians and Surgeons. His course was finally completed at Vienna and Berlin. Dr. Cooper selected Atlanta as his home, and early in his career established a lucrative practice and an enviable reputation. He was professor of obstetrics and gynecology of the Atlanta College of Physicians and Surgeons, gynecologist to the Wesley Memorial Hospital, and joint owner of the Elkin-Cooper Sanatorium on Luckie street.

The saddest feature of Dr. Cooper's death is that Mrs. Cooper and her children were not with him at the end. They have been traveling in Europe this summer, and although sent cablegram to return home they did not arrive until several days after his death. In addition to his wife and two children, Dr. Cooper is survived by his mother, Mrs. Mary P. Cooper, his brother, Thomas L. Cooper, and a sister, Mrs. Sarah Cooper Sanders.

"A good man, a physician measuring up to the highest ideals of his profession, a fine citizen, Dr. Cooper has been a potent factor in the life of Atlanta, and his death is a great loss to both city and State."

